# Synthesis, Biological Evaluation and 2D-QSAR Study of Halophenyl Bis-Hydrazones as Antimicrobial and Antitubercular Agents

**DOI:** 10.3390/ijms16048719

**Published:** 2015-04-20

**Authors:** Hatem A. Abdel-Aziz, Wagdy M. Eldehna, Mohamed Fares, Sara T. A. Al-Rashood, Khalid A. Al-Rashood, Marwa M. Abdel-Aziz, Dalia H. Soliman

**Affiliations:** 1 Department of Pharmaceutical Chemistry, College of Pharmacy, King Saud University, P.O. Box 2457, Riyadh 11451, Saudi Arabia; E-Mails: salrashood@ksu.edu.sa (S.T.A.A.-R.); krashood@ksu.edu.sa (K.A.A.-R.); 2 Department of Applied Organic Chemistry, National Research Centre, Dokki, Cairo 12622, Egypt; 3 Department of Pharmaceutical Chemistry, Faculty of Pharmacy, Egyptian Russian University, Badr City, Cairo 11829, Egypt; E-Mails: wagdy2000@gmail.com (W.M.E.); ph.fares@yahoo.com (M.F.); odihss2@gmail.com (D.H.S.); 4 The Regional Center for Mycology and Biotechnology, Al-Azhar University, Cairo 11759, Egypt; E-Mail: marwa2rcmb@yahoo.com; 5 Pharmaceutical Chemistry Department, Faculty of Pharmacy (Girls), Al-Azhar University, Cairo 11754, Egypt

**Keywords:** synthesis, antimicrobial activity, halophenyl bis-hydrazones, antimycobacterial, 2D-QSAR

## Abstract

In continuation of our endeavor towards the development of potent and effective antimicrobial agents, three series of halophenyl bis-hydrazones (**14a**–**n**, **16a**–**d**, **17a** and **17b**) were synthesized and evaluated for their potential antibacterial, antifungal and antimycobacterial activities. These efforts led to the identification of five molecules **14c**, **14g**, **16b**, **17a** and **17b** (MIC range from 0.12 to 7.81 μg/mL) with broad antimicrobial activity against *Mycobacterium tuberculosis*; *Aspergillus fumigates*; Gram positive bacteria, *Staphylococcus aureus*, *Streptococcus pneumonia*, and *Bacillis subtilis*; and Gram negative bacteria, *Salmonella typhimurium*, *Klebsiella pneumonia*, and *Escherichia coli*. Three of the most active compounds, **16b**, **17a** and **17b**, were also devoid of apparent cytotoxicity to lung cancer cell line A549. Amphotericin B and ciprofloxacin were used as references for antifungal and antibacterial screening, while isoniazid and pyrazinamide were used as references for antimycobacterial activity. Furthermore, three Quantitative Structure Activity Relationship (QSAR) models were built to explore the structural requirements controlling the different activities of the prepared bis-hydrazones.

## 1. Introduction

Despite the harmful impact of microbial threats to public health, large pharmaceutical companies have left the area of antimicrobial discovery and the number of scientists involved in the search for novel broad antimicrobial leads was reduced dramatically [1]. Unfortunately, the vast discoveries of new potent antimicrobial agents in the 1950s and 1960s led to over-confidence of eradication of the infectious diseases [2]. Moreover, only few classes of antibiotics (oxazolidinone, ketolide and lipoglycopeptide) were produced in the last 50 years, since the introduction of nalidixic acid in 1962. However, all of the antibacterial agents that have entered the market during this period were just modifications of existing molecules [2,3]. In the absence of an effective platform for antibiotic discovery [3] and overuse of antibiotics in humans and animals [4,5], bacteria exploited this opportunity by progressively developing resistance to most of the used antibiotics. Therefore, there is a great need to develop novel and effective antimicrobial and antimycobacterial drugs to combat this resistance.

Tuberculosis (TB) is a serious continuing major global health crisis and is ranked as the second leading cause of worldwide death among infectious diseases [6]. Nearly, 8.6 million people infected with *Mycobacterium tuberculosis* and 1.3 million died from the disease (including 320,000 deaths among HIV-positive people) in 2012, according to World Health Organization (WHO) global tuberculosis report 2013 [6]. The disease is aggravated by the worldwide continuous emergence of multidrug-resistant strains of *M. tuberculosis* (MDR-TB), extensively drug-resistant tuberculosis (XDR-TB) and totally drug-resistant tuberculosis (TDR-TB) [7,8]. The concern that tuberculosis may again become an incurable disease has increased nowadays, due to the magnitude and extent of drug-resistant strains [9,10]. Moreover, the longer duration of TB therapy and the increasing incidences of tuberculosis in immunocompromised patients emphasize the urgent need for discovery of new lead compounds to extend the range of effective TB treatment options [11,12,13].

Owing to their significant biological and pharmacological profiles, hydrazides and hydrazones have stood out as a prime scaffold for the discovery of innovative therapies. They were reported to possess antibacterial–antifungal [14,15], anticonvulsant [16], anti-inflammatory [17], antimalarial [18] and antitubercular activities [19,20,21,22]. Nifuroxazide, salinazid and verazide are examples of clinically approved hydrazone-containing antibacterial and antimycobacterial drugs [23,24]. Moreover, isonicotinoyl hydrazide (INH) and its isonicotinoyl hydrazone analogs, related to the hydrazide class, are well-established antitubercular agents [25,26,27]. INH also exhibits bacteriostatic effects on bacillus [28]. Additionally, several research groups have pointed out the importance of halogens incorporation in different scaffolds for the antitubercular activity enhancement [29,30]. In view of the antimicrobial property of the hydrazone pharmacophore, it was envisaged that its combined effect with an active moiety, such as benzothiazole and benzofuran of reported significant antitubercular activity [31,32,33,34], may result in increased antitubercular and antimicrobial activities.

With regard to anti-TB agents, and to antibacterials generally, the limitations of target-based screening approach as a tool of drug discovery paved the way for the resurgence of interest in the use of phenotypic screening in antimicrobial hits discovery [1]. So after extensive literature search and in continuation of our research work on hydrazides and hydrazones as antimicrobial agents [35,36], it was contemplated to synthesize some novel (hydrazono)propane hydrazonoyl chlorides (**14a**–**n**), ((hydrazono)-1-(phenylsulfonyl/tosyl)propan-2-ylidene) benzohydrazide/benzo[*d*]thiazole-2-carbohydrazides (**16a**–**d**) and ((hydrazono)-4-phenyl-1-(piperidin-1-yl)but-3-en-2-ylidene)benzohydrazide/benzofuran-2-carbohydrazides (**17a**,**b**), and evaluate their antimicrobial and antitubercular activity. In addition, we described three valid 2D-QSAR models in order to explore the structural requirements controlling these observed antibacterial and antitubercular activities.

## 2. Results and Discussion

### 2.1. Chemistry

**Scheme 1:**
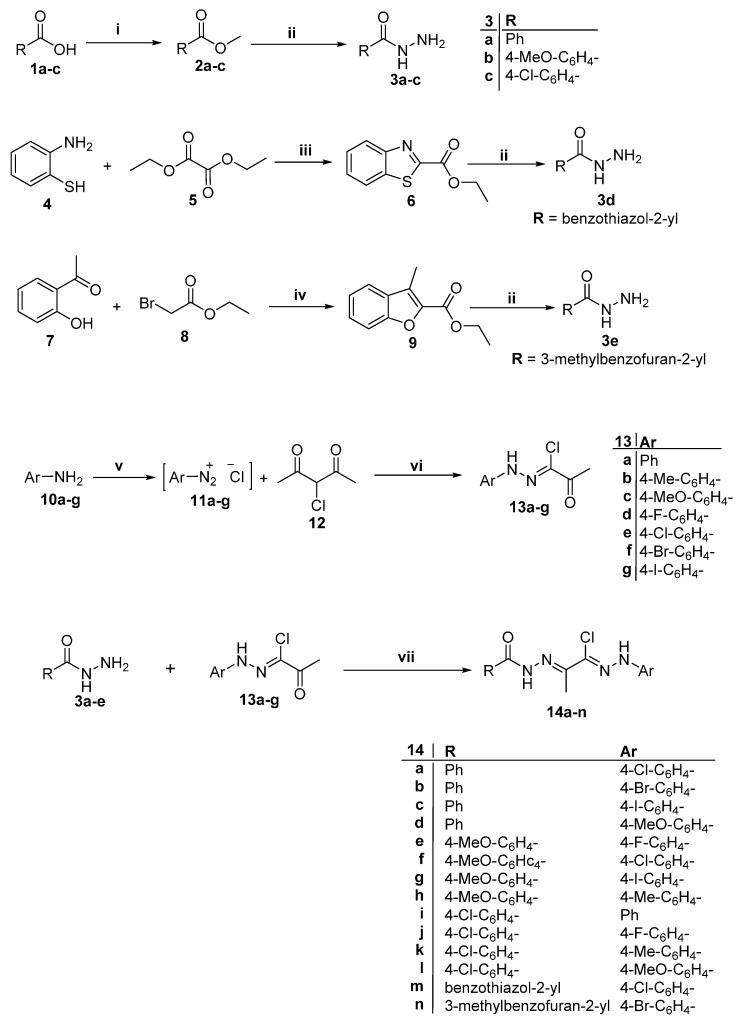
Synthesis of compounds **14a**–**n**. Reagents and conditions: **i**, MeOH/H_2_SO_4_/reflux 8 h; **ii**, NH_2_NH_2_.H_2_O/EtOH/reflux 4 h; **iii**, Neat, reflux 6 h; **iv**, MeOH/MeONa/reflux 8 h; **v**, HCl/NaNO_2_/H_2_O/0–5 °C; **vi**, CH_3_COONa/EtOH/0–5 °C; and **vii**, THF/reflux 10 h.

In an extension of our ongoing efforts towards developing potent antimicrobial agents [35,36,37,38,39], we synthesized eighteen novel bis-hydrazone derivatives bearing different aryl and heteroaryl rings. The synthetic route was initiated with the preparation of carbohydrazides **3a**–**c** [40], benzo[*d*]thiazole-2-carbohydrazide **3d** [35] and 3-methylbenzofuran-2-carbohydrazide **3e** [36] (Scheme 1). Diazodization of aromatic amines **10a**–**g** with hydrochloric acid and sodium nitrite gave diazonium salts **11a**–**g**, which were subsequently coupled with 3-chloropentane-2,4-dione **12** in ethanolic sodium acetate (Japp-Klingemann reaction) and afforded oxo-*N*-arylpropanehydrazonoyl chlorides **13a**–**g**, respectively [41] (Scheme 1). The reaction of carbohydrazides **3a**–**e** with 2-oxo-*N*'*-*(4substitutedphenyl)propanehydrazonoyl chloride **13a**–**g** in refluxing tetrahydrofuran (THF) afforded bis-hydrazones **14a**–**n** (Scheme 1).

The Fourier transform infrared spectroscopy (FTIR) of bis-hydrazones **14a**–**l** showed the presence of stretching vibrations of carbonyl group in the region 1667–1683 cm^−1^ in addition to the absorption bands of two NH functions in the region 3167–3404 cm^−1^. In the ^1^H NMR spectra two NH groups showed two D_2_O exchangeable signals in the regions δ: 10.00–10.27 and δ: 10.64–10.90 ppm, in addition to the singlet signal of the methyl group in the region δ: 2.29–2.38 ppm. Recently, we reported the X-ray diffraction structure for an analogue of compounds **14a**–**n**, which confirmed the (1*Z*,2*E*)-configuration of these bis-hydrazones in solid state [36].

**Scheme 2:**
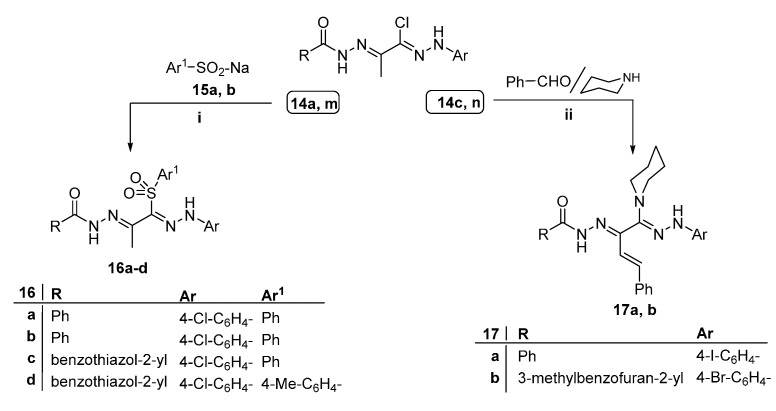
Synthesis of **16a**–**d** and **17a**,**b**. Reagents and conditions: **i**, EtOH/reflux 10 h; and **ii**, EtOH/reflux 15 h.

Arylsulfones are a promising class of antimicrobial agents [42,43]. To explore the influence of incorporating the sulfone moiety in the bis-hydrazone scaffold on the antimicrobial activity, some derivatives of the first series **14a** and **14m** have been selected for the synthesis of new bis-hydrazones bearing a phenylsulfone and tosyl moieties. Thus, reaction of **14a** or **14m** with sodium benzenesulfinate **15a** or sodium *p*-methylbenzenesulfinate **15b** afforded the corresponding sulfones **16a**–**d**, respectively (Scheme 2). The IR spectra of **16a**–**d** exhibited characteristic absorption band at 1639–1684 cm^−1^ due to acetyl C=O, while that of the sulfonyl functionality was observed in the regions 1134–1137 and 1247–1290 cm^−1^. Their ^1^H NMR spectra exhibited the two D_2_O-exchangeable signal of hydrazone NH at δ 11.67–13.39 ppm for =NNH– and δ 14.17–14.58 ppm for –CONH–. The compounds **16a** and **16d** showed characteristic ^13^C NMR signals at 15.06 and 13.00 ppm corresponding to the carbon of methyl groups and signals at 165.28 and 167.35 ppm attributed to the carbons of carbonyl function, respectively.

The formation of piperidine derivatives **17a** and **17b** was accomplished by heating each of bis-hydrazones **14c** or **14n** with piperidine in ethanol, which resulted in elimination of hydrogen chloride and the formation of a single product, in each case. The ^1^H NMR of **17a** and **17b** revealed the protons of piperidine moiety in the regions δ 1.56–1.64 and 3.21–3.28 ppm while the ^13^C NMR of **17b** showed the signals of five sp3 carbons at around 23.95 and 46.60 ppm, respectively.

### 2.2. Anti-Microbial Activity

Antibacterial and antifungal activities were performed at the Regional Center for Mycology and Biotechnology (RCMB), Al-Azhar University, Cairo, Egypt. Initially, target compounds **14a**–**n** and reference drugs were evaluated *in vitro* for their antibacterial and antifungal activity, by inhibition zone technique and minimum inhibitory concentration (MIC), using one fungi: *A. fumigatus* (RCMB 02568), three Gram positive bacteria: *S. aureus* (RCMB 010028) *S. pneumonia* (RCMB 010010) and *B. subtilis* (RCMB 010069), four Gram-negative bacteria: *P. aeruginosa* (RCMB 010043), *S. typhimurium* (RCMB 010315), *K. pneumonia* (RCMB 0010093) and *E. coli* (RCMB 010052) with the addition of *M. tuberculosis* (RCMB 010126), For the optimization purpose, the inactive and moderate active agents, **14a**, **14m** and **14n** were selected for further modification, hoping to increase the antimicrobial as well as the antimycobacterial activities. Compounds **14a** and **14m** modified to the sulfone derivatives **16a**, **16b** and **16c**, **16d**, respectively, while compound **14n** optimized to the piperidine derivative **17b**. Encouraged by the promising results of these modified analogs, the most active compound **14c** was further modified to the piperidine counterpart **17a**.

#### 2.2.1. Anti-Fungal Activity

*Aspergillus fumigatus* is largely responsible for increased the incidence of invasive aspergillosis (IA) in immunocompromised patients [44]. Moreover, the mortality rate due to invasive fungal diseases is still unacceptable high, because of a limited number of antifungal agents [45].

Data in Table 1 and Table 2 revealed that compounds **14c**, **14e**, **14g**, **16b**, **17a** and **17b** showed a remarkable activity against *Aspergillus fumigatus*.

**Table 1:** Antimicrobial activities of the synthesized bis-hydrazones against the pathological organisms expressed as inhibition diameter zones in millimeters (mm) based on well diffusion assay. 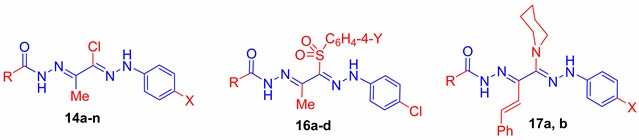

Comp.	*R*	*X* or *Y*	Fungi	Gram Positive Bacteria			Gram Negative Bacteria			
			Af	Sa	Sp	Bs	Pa	St	Kp	Ec
**14a**	Ph-	Cl	NA	NA	NA	NA	NA	NA	NA	NA
**14b**	Ph-	Br	NA	13.9 ± 0.44	12.4 ± 0.63	14.8 ± 0.58	NA	15.1 ± 0.58	16.4 ± 0.72	10.4 ± 0.44
**14c**	Ph-	I	21.2 ± 0.25	21.9 ± 0.44	23.2 ± 0.37	23.9 ± 0.25	17.6 ± 0.25	20.4 ± 0.63	19.2 ± 0.25	20.7 ± 0.63
**14d**	Ph-	OMe	11.2 ± 0.32	9.4 ± 0.58	11.4 ± 0.72	13.2 ± 0.72	NA	11.6 ± 0.25	13.7 ± 0.44	10.2 ± 0.58
**14e**	4-MeOC_6_H_4_-	F	19.7 ± 0.32	20.1 ± 0.58	20.9 ± 0.58	20.2 ± 0.32	NA	21.3 ± 0.58	19.2 ± 0.24	20.4 ± 0.58
**14f**	4-MeOC_6_H_4_-	Cl	16.9 ± 0.58	17.1 ± 0.58	18.8 ± 0.44	20.6 ± 0.44	NA	17.8 ± 0.63	16.4 ± 0.37	18.5 ± 0.58
**14g**	4-MeOC_6_H_4_-	I	21.4 ± 0.44	20.8 ± 0.58	22.3 ± 0.63	23.2 ± 0.58	15.3 ± 0.25	22.4 ± 0.44	19.9 ± 0.25	21.3 ± 0.44
**14h**	4-MeOC_6_H_4_-	Me	15.6 ± 0.25	17.2 ± 0.44	17.8 ± 0.37	19.9 ± 0.25	NA	20.3 ± 0.63	18.6 ± 0.25	19.7 ± 0.63
**14i**	4-ClC_6_H_4_-	H	NA	NA	NA	NA	NA	NA	NA	NA
**14j**	4-ClC_6_H_4_-	F	NA	NA	NA	NA	NA	NA	NA	NA
**14k**	4-ClC_6_H_4_-	Me	15.7 ± 0.58	16.9 ± 0.37	15.6 ± 0.25	17.2 ± 0.58	NA	19.1 ± 0.63	16.5 ± 0.63	18.1 ± 0.72
**14l**	4-ClC_6_H_4_-	OMe	15.3 ± 0.25	18.4 ± 0.58	16.2 ± 0.63	17.3 ± 0.58	NA	17.9 ± 0.58	18.2 ± 0.72	15.8 ± 0.63
**14m**	Benzothiazol-2-yl	Cl	NA	16.2 ± 0.63	14.7 ± 0.44	15.3 ± 0.44	NA	18.6 ± 0.58	19.8 ± 0.58	18.6 ± 0.44
**14n**	3-Methylbenzofuran-2-yl	Br	NA	NA	NA	NA	NA	NA	NA	NA
**16a**	Ph-	H	14.3 ± 0.37	17.2 ± 0.63	16.8 ± 0.44	18.3 ± 0.44	NA	16.8 ± 0.25	13.4 ± 0.44	15.2 ± 0.63
**16b**	Ph-	Me	21.2 ± 0.58	22.3 ± 0.63	22.8 ± 0.25	24.2 ± 0.25	18.9 ± 0.25	22.9 ± 0.37	21.4 ± 0.58	20.7 ± 0.63
**16c**	Benzothiazol-2-yl	H	18.1 ± 0.58	14.8 ± 0.58	13.8 ± 0.58	16.3 ± 0.44	NA	19.8 ± 0.25	18.7 ± 0.44	16.1 ± 0.63
**16d**	Benzothiazol-2-yl	Me	19.1 ± 0.63	15.7 ± 0.15	17.3 ± 0.18	19.8 ± 0.22	NA	19.7 ± 0.48	16.5 ± 0.37	17.6 ± 0.25
**17a**	Ph-	I	20.6 ± 0.58	21.3 ± 0.58	21.9 ± 0.44	23.1 ± 0.44	17.2 ± 0.58	21.3 ± 0.58	20.4 ± 0.37	20.6 ± 0.58
**17b**	3-Methylbenzofuran-2-yl	Br	20.4 ± 0.58	20.9 ± 0.44	21.3 ± 0.58	21.9 ± 0.36	19.1 ± 0.44	22.4 ± 0.58	21.4 ± 0.19	22.9 ± 0.58
**AB**			**19.5 ± 0.21**	nt	nt	nt	nt	nt	nt	nt
**CF**			nt	**20.0 ± 0.34**	**20.3 ± 0.58**	**20.4 ± 0.14**	**19.2 ± 0.15**	**19.8 ± 0.63**	**19.7 ± 0.12**	**20.3 ± 0.44**

NA: No Activity; The screening organisms, Mould: *Aspergillus fumigatus* (RCMB 02568, Af); Gram positive bacteria: *Staphylococcus aureus* (RCMB 010028, Sa), *Streptococus pneumoniae* (RCMB 010010, Sp), and *Bacillus subtilis* (RCMB 010069, Bs); Gram negative bacteria: *Pseudomonas aeruginosa* (RCMB 010043, Pa), *Salmonella typhimurium* (RCMB 010315, St), *Klebsiella penumoniae* (RCMB 0010093, Kp) and *Escherichia coli* (RCMB 010052, Ec); AB: Amphotericin B; CF: Ciprofloxacin; Comp.: Compound; nt: not tested; Results shown in bold letters indicate more antituberculosis activity of compounds compared to others.

**Table 2:** Antimicrobial activities of the tested standards and synthesized compounds as MICs (μg/mL).

Comp.	Fungi	Gram Positive Bacteria			Gram Negative Bacteria			
	Af	Sa	Sp	Bs	Pa	St	Kp	Ec
**14a**	>125	>125	>125	>125	>125	>125	>125	>125
**14b**	>125	125	125	62.50	>125	62.50	31.25	125
**14c**	**0.98**	**0.49**	**0.24**	**0.12**	7.81	**1.95**	**3.90**	**0.98**
**14d**	>125	>125	125	125	>125	125	125	125
**14e**	**1.95**	**1.95**	**0.98**	**1.95**	>125	**0.98**	**3.90**	**1.95**
**14f**	15.63	15.63	3.90	**0.98**	>125	7.81	15.63	3.90
**14g**	**0.98**	**0.98**	**0.49**	**0.24**	62.50	**0.49**	**1.95**	**0.98**
**14h**	31.25	31.25	7.81	**1.95**	>125	**1.95**	**3.90**	**1.95**
**14i**	>125	>125	>125	>125	>125	>125	>125	>125
**14j**	>125	>125	>125	>125	>125	>125	>125	>125
**14k**	31.25	15.63	31.25	15.63	>125	**3.90**	15.63	7.81
**14l**	62.50	7.81	31.25	15.63	>125	7.81	7.81	31.25
**14m**	>125	31.25	62.50	62.50	>125	**3.90**	**1.95**	3.90
**14n**	>125	>125	>125	>125	>125	>125	>125	>125
**16a**	125	15.63	15.63	7.81	>125	15.63	125	62.50
**16b**	**0.49**	**0.49**	**0.24**	**0.12**	**3.90**	**0.24**	**0.98**	**0.98**
**16c**	7.81	62.50	125	31.25	>125	**3.90**	**3.90**	31.25
**16d**	3.90	31.25	15.63	**1.95**	>125	**3.90**	15.63	7.81
**17a**	**0.98**	**0.98**	**0.49**	**0.24**	15.63	**0.98**	**1.95**	**0.98**
**17b**	**1.95**	**0.98**	**0.98**	**0.98**	**3.90**	**0.49**	**0.98**	**0.24**
**AB**	1.95	nt	nt	nt	nt	nt	nt	nt
**CF**	nt	1.95	1.95	1.95	3.90	3.90	3.90	1.95

The screening organisms, Mould: *Aspergillus fumigatus* (RCMB 02568, An); Gram positive bacteria: *Staphylococcus aureus* (RCMB 010028, Sa), *Streptococus pneumoniae* (RCMB 010010, Sp), and *Bacillus subtilis* (RCMB 010069, Bs); Gram negative bacteria: *Pseudomonas aeruginosa* (RCMB 010043, Pa), *Salmonella typhimurium* (RCMB 010315, St), *Klebsiella penumoniae* (RCMB 0010093, Kp) and *Escherichia coli* (RCMB 010052, Ec); AB: Amphotericin B; CF: Ciprofloxacin; Comp.: Compound; nt: not tested; Results shown in bold letters indicate more or equal activity of the compounds compared to the standard drugs.

The sulfone derivative **16b** (MIC = 0.49 μg/mL) exhibited the highest potency against *Aspergillus fumigatus* organism where it was four times more active than the reference drug Amphotericin B (MIC = 1.95 μg/mL), a membrane-active polyene macrolide antibiotic and an antifungal compound [46]. Para-iodo substituted members; **14c**, **14g** and **17a** revealed twofold increase in activity than the reference drug with MIC = 0.98 μg/mL. Compounds **14e** and **17b** were equipotent to the Amphotericin B.

#### 2.2.2. Antibacterial Activity

As indicated in Table 1 and Table 2, compounds **14c**, **14e**, **14g**, **16b** and **17a** and **17b** displayed broad-spectrum antibacterial activity against the tested Gram positive and Gram negative bacteria as compared with ciprofloxacin. Ciprofloxacin is a broad spectrum widely used antibacterial and is considered the most potent marketed fluoroquinolone against gram-negative bacteria [47].

Regarding Gram positive bacteria, compounds **14c** and **16b** emerged as the most potent analogs. There were found to be fourfold more active against *S. aureus*, eightold more active than *S. pneumonia* and 16 times more active than *B. subtilis* compared to the standard drug (MIC = 1.95 μg/mL), while **14g** and **17a** showed twofold increased inhibition against *S. aureus*, fourfold against *S. pneumonia* and eightfold against *B. subtilis*. Compound **17b** showed double the activity of ciprofloxacin against the selected Gram positive bacteria. On the other hand, the counterparts, **14e** and **14f** also displayed double the activity of the standard drug against *S. pneumonia* and *B. subtilis*, respectively. Moreover, **14e** was equipotent to ciprofloxacin against *S. aureus*, compounds **14e**, **14h** and **16d** were also equipotent to the standard drug against *B. subtilis.* Rest of the compounds elicited moderate to fair activity towards Gram positive bacteria.

Concerning Gram negative bacteria, **16b** and **17b** counterparts (MIC = 3.90 μg/mL) were equipotent to the ciprofloxacin in inhibiting the growth of *P. aeruginosa*. All the compounds were screened against *Salmonella typhimurium* to evaluate their anti-typhoid activity. Compound **16b** was the most active against *S. Typhimurium* as it exhibited 16-fold increased activity than ciprofloxacin (MIC = 3.90 μg/mL). Moreover, compounds **14g** and **17b** were eightfold, **14e** and **17a** were fourfold while **14c** and **14h** were twofold more potent than the standard drug. Furthermore, **14k**, **14m**, **16c** and **16d** counterparts were equipotent to the reference drug in inhibiting *S. Typhimurium*.

The activity of all compounds against *K. pneumonia* was investigated. Highest activity observed in **16b** and **17b** counterparts, followed by **14g**, **14m** and **17a**.

Compound **17b** showed the best activity in inhibiting *E. coli*, whereas, **14c**, **14g**, **16b** and **17a** showed good activity. Finally, **14c**, **14e**, **14h** and **16c** were equipotent to ciprofloxacin in inhibiting *K. pneumonia*, while, compounds **14e** and **14h** were also equipotent to the reference drug against *E. coli*.

#### 2.2.3. Antimycobacterial Activity

By investigating the antitubercular activity of a limited focused library of twenty bis-hydrazone derivatives **14a**–**n**, **16a**–**d**, **17a** and **17b** (Table 3), it was observed that, in general, best activity is found in compounds of higher lipophilicity, as observed by Maccari *et al.* [48].

Regarding **14a**–**n** series, compounds **14c** and **14g** showed a significant antimycobacterial activity (MIC = 7.81 μg/mL). This indicated that the improvement in the activity is linked to the para substitution of iodo-atom linked to *N*(1) in the hydrazonoyl chloride moiety and the presence of unsubstituted or *p*-methoxy phenyl at the carbonyl group. Concerning the sulfone derivatives **16a**–**d** and the piperidine analogs **17a** and **17b**, the antimycobacterial activity was observed in **16b**, **17a** and **17b** with MIC ranged from 1.95–3.90 μg/mL relative to the reference drugs Isoniazide (MIC = 0.40 μg/mL) and Pyrazinamide (MIC = 3.21 μg/mL). The preliminary antitubercular results afford an excellent lead for further development of these molecules as novel antimycobacterial agents.

**Table 3:** Anti-tubercular activities and C log*P* measurements of bis-hydrazones.

Comp.	I.Z	MIC	C Log*P* ^a^
**14a**	NA	NA	5.615
**14b**	NA	NA	5.746
**14c**	18.3 ± 0.25	**7.81**	6.02
**14d**	NA	NA	4.994
**14e**	NA	NA	5.157
**14f**	NA	NA	5.672
**14g**	18.4 ± 0.25	**7.81**	6.077
**14h**	NA	NA	5.442
**14i**	NA	NA	5.615
**14j**	NA	NA	5.779
**14k**	NA	NA	6.064
**14l**	NA	NA	5.672
**14m**	NA	NA	5.780
**14n**	NA	NA	6.687
**16a**	NA	NA	5.504
**16b**	19.4 ± 0.25	**3.90**	5.952
**16c**	NA	NA	5.669
**16d**	11.3 ± 0.37	125	6.118
**17a**	19.3 ± 0.37	**3.90**	8.283
**17b**	20.2 ± 0.19	**1.95**	8.69
**Isoniazide**	nt	0.40	−0.969
**Pyrazinamide**	nt	3.21	−0.711

^a^ Calculated by http://www.molinspiration.com/; NA: No Activity; MIC: Minimum Inhibition Zone; Comp.: Compound; I.Z.: Inhibition Zone; nt: not tested; Results shown in bold letters indicate more antituberculosis activity of compounds compared to others.

#### 2.2.4. *In Vitro* Cytotoxicity

*In vitro* cytotoxicity of most active antimicrobial and antitubercular compounds **16b**, **17a** and **17b** was evaluated against human lung cancer cell line (A-549) using SRB method. Doxorubicin was used as the reference drug. The IC_50_ values obtained for these compounds are listed in Table 4. None of the tested compounds displayed any significant cytotoxicity against lung cells (A549), thereby providing a high therapeutic index.

**Table 4:** Levels of cytotoxicity induced by selected compounds on A549 cells.

Compound	IC_50_ (μM)
**16b**	75.3
**17a**	>100
**17b**	>100
**Doxorubicin**	2.3

### 2.3. 2D QSAR Study

#### 2.3.1. Development of QSAR Models

QSAR analyses for antibacterial and antimycobacterial activities of the prepared derivatives (**14a**–**n**, **16a**–**d**, **17a** and **17b**) were performed in order to correlate the biochemical data with the synthesized compounds, and to identify positive and negative structural features within the three series. The analysis was run by means of the DS 2.5 software (Discovery Studio 2.5, Accelrys, Co., Ltd., San Diego, CA, USA).

A set of the newly synthesized bis-hydrazones (**14a**–**n**, **16a**–**d**, **17a** and **17b**) was used as a training set with their measured pMIC against *Bacillis subtilis*, *Klebsiella pneumonia* and *Mycobacterium tuberculosis* for QSAR modeling. “Calculate Molecular Properties” module was used for calculating different molecular properties for the training set compounds. 2D Descriptors involved: AlogP, molecular properties, molecular property counts, surface area and volume and topological descriptors, while the 3D descriptors involved: Dipole, jurs descriptors, principle moments of inertia, shadow indices and surface area and volume. Genetic function approximation (GFA) was utilized to search for the best possible QSAR regression equation capable of correlating the variations in the biological activities of the training compounds with variations in the generated descriptors, *i.e.*, multiple linear regression modeling (MLR) [49]. QSAR model was validated employing leave one-out cross-validation by setting the folds to a number much larger than the number of samples, *r*^2^ (squared correlation coefficient value) and *r*^2^ prediction (predictive squared correlation coefficient value), residuals between the predicted and experimental activity of the test set and training set.

#### 2.3.2. QSAR Study Results

Equation (1). Represents the best performing QSAR model for the activity against *Bacillis subtilis*;

(1) −logMIC = −17.1387 − 0.2620 Jurs_WPSA_3 + 1.7027 Shadow_nu + 1.4314 Shadow_Ylength

Equation (2). Represents the best performing QSAR model for the activity against *Klebsiella pneumonia*;

(2) −logMIC = 2.6641 + 0.0174 Jurs_WNSA_1 + 0.1523 Shadow_YZ − 2.13292 Shadow_Zlength

Equation (3). Represents the best performing QSAR model for the antimycobacterial activity;

(3) −logMIC= −7.224 − 0.0015 Jurs_PPSA_2 + 0.6156 Shadow_Ylength

**Figure 1:**
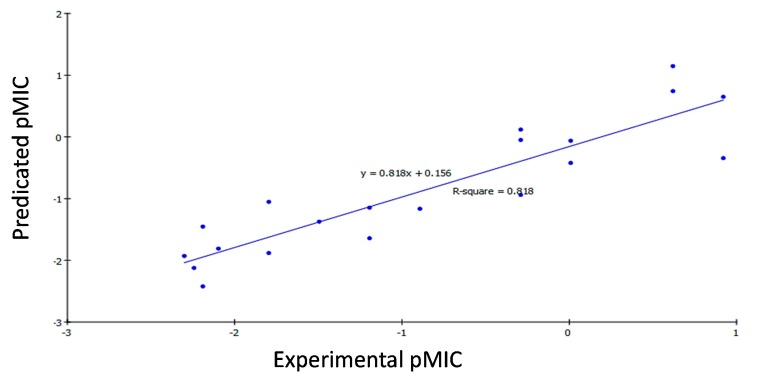
Predicted *versus* experimental pMIC of the tested compounds against *Bacillis subtilis* according to Equation (1) (*r*^2^ = 0.818).

**Figure 2:**
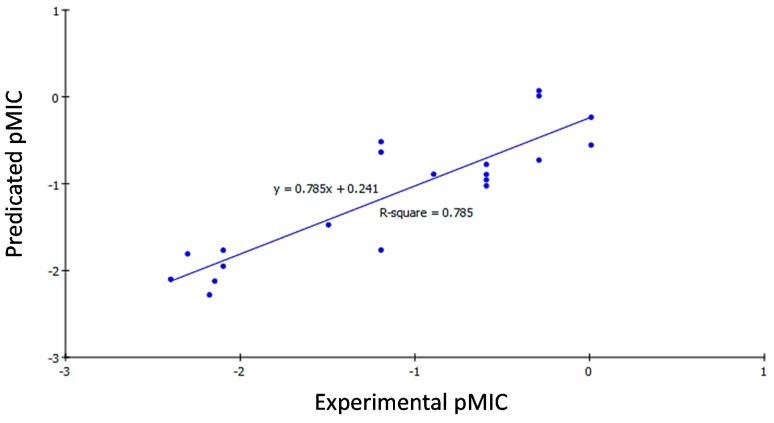
Predicted *versus* experimental pMIC of the tested compounds against *Klebsiella pneumonia* according to Equation (2) (*r*^2^ = 0.785).

**Figure 3:**
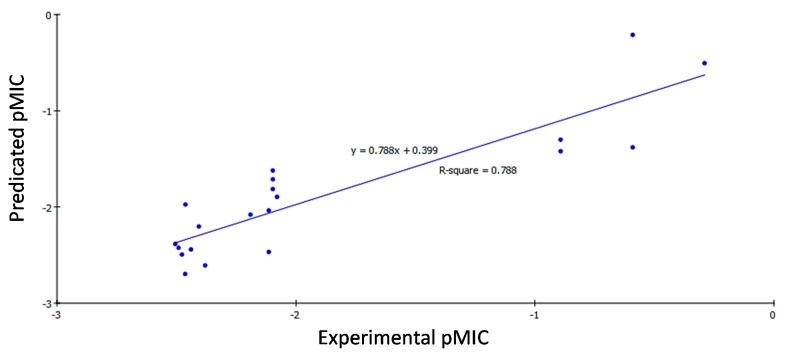
Predicted *versus* experimental pMIC of the tested compounds against *Mycobacterium tuberculosis* according to Equation (3) (*r*^2^ = 0.788).

According to Equations (1)–(3), the QSAR models were represented graphically by scattering plots of the experimental *versus* the predicted bioactivity values −logMIC for the training set compounds as shown in Figure 1, Figure 2 and Figure 3. The method used to build the model was Least-Squares, *r*^2^ = 0.818, 0.785 and 0.788, respectively, *r*^2^ (adj) = 0.784, 0.745 and 0.763, respectively, *r*^2^ (pred) = 0.712, 0.665 and 0.684, respectively, Least-Squared error = 0.2244, 0.1403 and 0.1148, respectively, where *r*^2^ (adj) is *r*^2^ adjusted for the number of terms in the model; *r*^2^ (pred) is the prediction *c*, equivalent to *q*^2^ from a leave-1-out cross validation.

In conclusion, Equations (1)–(3) suggested that the antibacterial activities of the synthesized compounds are mainly affected by jurs descriptors and shadow indices. Jurs descriptors are those ones that combine shape and electronic information to characterize molecules [50].

The descriptors are calculated by mapping atomic partial charges on solvent-accessible surface areas of individual atoms. Jurs_WPSA_3 and Jurs_WNSA_1 are the surface-weighted charged partial surface areas “set of six descriptors (Jurs_WPSA_1, Jurs_WPSA_2, Jurs_WPSA_3, Jurs_WNSA_1, Jurs_WNSA_2 and Jurs_WNSA_3) obtained by multiplying descriptors 1 to 6 by the total molecular solvent-accessible surface area and dividing by 1000”. Jurs_PPSA_2 is atomic charge weighted positive surface area “Sum of the product of solvent-accessible surface area × partial charge for all positively charged atoms”. In addition, shadow indices are set of geometric descriptors to characterize the shape of the molecules [51].

The descriptors are calculated by projecting the model surface on three mutually perpendicular planes: *xy*, *yz*, and *xz*. These descriptors depend not only on conformation, but also on the orientation of the model. To calculate them, the models are first rotated to align the principal moments of inertia with the *x*-, *y*-, and *z*-axes. Shadow_YZ is area of the molecular shadow in the *yz* plane, Shadow_nu is ratio of largest to smallest dimension, Shadow_Ylength is Length of molecule in the *y* dimension while, Shadow_Zlength is Length of molecule in the *z* dimension.

#### 2.3.3. QSAR Validation

Robustness of the established QSAR models (1, 2 and 3) was verified by using; Leave-one-out (LOO) internal validation (*r*^2^ = 0.818, 0.785 and 0.788, respectively). Cross-validation was also employed where *q*^2^, which is equivalent to *r*^2^ (pred), was 0.712, 0.665 and 0.684, respectively. In addition, validation was employed by measuring the residuals between the experimental and the predicted activities of the training set (Table 5). Interestingly, the predicted activities by the QSAR models were very close to those experimentally observed, indicating that these models could be applied for prediction of more effective hits having the same skeletal framework.

**Table 5:** Experimental activities of the synthesized derivatives against the predicted activity according to Equations (1)–(3).

Comp.	*Bacillis subtilis*			*Klebsiella pneumonia*			*Mycobacterium tuberculosis*		
	Experimental Activity (pMIC)	Predicted Activity (pMIC)	Residual	Experimental Activity (pMIC)	Predicted Activity (pMIC)	Residual	Experimental Activity (pMIC)	Predicted Activity (pMIC)	Residual
**14a**	−2.1903	−1.4532	−0.7371	2.3010	−1.8082	−0.4929	−2.0792	−1.9173	−0.1619
**14b**	−1.7959	−1.0525	−0.7434	−1.4949	−1.4739	−0.0209	−2.0969	−1.7353	−0.3616
**14c**	0.9208	−0.3440	1.2648	−0.5911	−1.0238	0.4328	−0.8927	−1.4641	0.5715
**14d**	−2.0969	−1.8098	−0.2871	−2.0969	−1.9502	−0.1467	−2.4771	−2.5540	0.0769
**14e**	−0.2900	−0.9401	0.6501	−0.5911	−0.9562	0.3651	−2.4065	−2.2571	−0.1494
**14f**	0.0088	−0.4222	0.4309	−1.1940	−0.5166	−0.6774	−2.4624	−1.9998	−0.4626
**14g**	0.6198	1.1471	−0.5273	−0.2900	0.0707	−0.3607	−0.8927	−1.3632	0.4706
**14h**	−0.2900	0.1190	−0.4091	−0.5911	−0.7779	0.1869	−2.0969	−1.9088	−0.1881
**14i**	−2.3010	−1.9291	−0.3719	−2.3979	−2.1004	−0.2975	−2.5052	−2.3546	−0.1505
**14j**	−2.2430	−2.1235	−0.1195	−2.1461	−2.1224	−0.0237	−2.4393	−2.3799	−0.0595
**14k**	−1.1940	−1.6423	0.4483	−1.1940	−1.7642	0.5703	−2.4914	−2.3827	−0.1087
**14l**	−1.1940	−1.1464	−0.0475	−0.8927	−0.8912	−0.0015	−2.4639	−2.6564	0.1925
**14m**	−1.7959	−1.8821	0.0863	−0.2900	−0.7286	0.4386	−2.3802	−2.5155	0.1352
**14n**	−2.1903	−2.4221	0.2318	−2.1761	−2.2809	0.1048	−2.1139	−2.4051	0.2912
**16a**	−0.8927	−1.1653	0.2727	−2.0969	−1.7667	−0.3302	−2.1139	−2.1128	−0.0012
**16b**	0.9208	0.6497	0.2711	0.0088	−0.5553	0.5641	−0.5911	−1.4733	0.8823
**16c**	−1.4949	−1.3730	−0.1218	−0.5911	−0.8948	0.3037	−2.1903	−2.0308	−0.1595
**16d**	−0.2900	−0.0496	−0.2405	−1.1940	−0.6373	−0.5567	−2.0969	−1.5745	−0.5225
**17a**	0.6198	0.7416	−0.1218	−0.2900	0.0110	−0.3010	−0.5911	−0.2366	−0.3545
**17b**	0.0088	−0.0622	0.0710	0.0088	−0.2344	0.2432	−0.2900	−0.3499	0.0599

## 3. Experimental Section

### 3.1. Chemistry

Melting points were measured with a Stuart melting point apparatus and were uncorrected. The NMR spectra were recorded by Varian Gemini-300BB 300 MHz FT-NMR spectrometers (Varian Inc., Palo Alto, CA, USA). ^1^H and ^13^C spectra were run at 300 and 75 MHz, respectively, in deuterated dimethylsulfoxide (DMSO-*d*_6_). Chemical shifts (δ_H_) are reported relative to TMS as internal standard. All coupling constant (*J*) values are given in hertz. Chemical shifts (δ_C_) are reported relative to DMSO-*d*_6_ as internal standards. The abbreviations used are as follows: s, singlet; d, doublet; m, multiplet. IR spectra were recorded with a Bruker FT-IR spectrophotometer. Electron impact mass spectra were measured on a Varian MAT 311-A (70 Electronvolt) Reaction courses and product mixtures were routinely monitored by thin layer chromatography (TLC) on silica gel precoated F_254_ Merck plates. Unless otherwise noted, all solvents and reagents were commercially available and used without further purification.

#### 3.1.1. Synthesis of Hydrazones **14a**–**n**

To a stirred solution of the appropriate carbohydrazides **3a**–**e** (5 mmol), 2-oxo-*N*'-(4-substitutedphenyl)propanehydrazonoyl chloride **13a**–**g** (5 mmol) was added in THF (30 mL). The reaction mixture was heated under reflux for 10 h. The solid product obtained upon cooling was filtered off and recrystallized from dioxane to afford the corresponding hydrazones **14a**–**n** with 65%–80% yield.

##### (1*Z*,2*E*)-2-(2-Benzoylhydrazono)-*N*-(4-chlorophenyl)propanehydrazonoyl chloride (**14a**)

Yellow powder (yield 75%), melting point (m.p.): 199–200 °C; IR (KBr, *ν* cm^−1^): 3303, 3353 (2NH), 1643 (C=O) and 1588 (C=N); ^1^H NMR (DMSO-*d*_6_) δ ppm: 2.38 (s, 3H, CH_3_), 7.31–7.37 (m, 4 H, Ar–H), 7.49–7.59 (m, 3H, Ar–H), 7.87 (d, 2H, *J* = 6.9 Hz, Ar–H), 10.27 (s, D_2_O exchangeable (exch.), 1H, =NNH–), 10.84 (s, D_2_O exch., 1H, –CONH–);^13^C NMR (DSMO-*d*_6_) δ ppm: 16.50, 115.33, 124.50, 124.57, 128.31, 128.95, 131.52, 133.72, 135.50, 137.00, 142.52, 155.00; MS *m*/*z* [%]: 350 [(M + 2)^+^, 4.7], 348 [M^+^, 7.5], 105 [100].

##### (1*Z*,2*E*)-2-(2-Benzoylhydrazono)-*N*-(4-bromophenyl)propanehydrazonoyl chloride (**14b**)

Yellow powder (yield 78%), m.p.: 197–199 °C; IR (KBr, *ν* cm^−1^): 3352, 3302 (2NH), 1641 (C=O) and 1583 (C=N); ^1^H NMR (DMSO-*d*_6_) δ ppm: 2.38 (s, 3H, CH_3_), 7.28 (d, 2H, *J* = 9.0 Hz, H-2 and H-6 of 4-BrC_6_H_4_), 7.43 (d, 2H, *J* = 9.0 Hz, H-3 and H-5 of 4-BrC_6_H_4_), 7.48–7.59 (m, 3H, H-3, H-4 and H-5 of –C_6_H_5_), 7.87 (d, 2H, *J* = 8.1 Hz, H-2 and H-6 of –C_6_H_5_), 10.27 (s, D_2_O exch., 1H, =NNH–), 10.84 (s, D_2_O exch., 1H, –CONH–); MS *m*/*z* [%]: 396 [(M + 2)^+^, 1.2], 394 [M^+^, 4.5], 105 [100].

##### (1*Z*,2*E*)-2-(2-Benzoylhydrazono)-*N*-(4-iodophenyl)propanehydrazonoyl chloride (**14c**)

Brown powder (yield 80%), m.p.: 213–215 °C; IR (KBr, *ν* cm^−1^): 3315, 3283 (2NH), 1639 (C=O) and 1560 (C=N); ^1^H NMR (DMSO-*d*_6_) δ ppm: 2.37 (s, 3H, CH_3_), 7.16 (d, 2H, *J* = 8.7 Hz, H-2 and H-6 of 4-IC_6_H_4_), 7.48–7.56 (m, 3H, H-3, H-4 and H-5 of C_6_H_5_), 7.58 (d, *J* = 8.7 Hz, 2H, H-3 and H-5 of 4-IC_6_H_4_), 7.87 (d, 2H, *J* = 6.9 Hz, H-2 and H-6 of C_6_H_4_), 10.25 (s, D_2_O exch., 1H, =NNH–), 10.84 (s, D_2_O exch., 1H, –CONH–); MS *m*/*z* [%]: 442 [(M + 2)^+^, 0.8], 440 [M^+^, 2.5], 404 [100].

##### (1*Z*,2*E*)-2-(2-Benzoylhydrazono)-*N*-(4-methoxyphenyl)propanehydrazonoyl chloride (**14d**)

Yellow powder (yield 65%), m.p.: 189–192 °C; IR (KBr, *ν* cm^−1^): 3316, 3291 (2NH), 1645 (C=O) and 1563 (C=N); ^1^H NMR (DMSO-*d*_6_) δ ppm: 2.37 (s, 3H, CH_3_), 3.72 (s, 3H, OCH_3_), 6.88 (d, 2H, *J* = 9 Hz, H-3 and H-5 of 4-OCH_3_C_6_H_4_), 7.26 (d, 2H, *J* = 9 Hz, H-2 and H-6 of 4-OCH_3_C_6_H_4_), 7.48 (t, 1H, *J* = 7.4 Hz, H-4 of C_6_H_5_), 7.56 (d, 2H, *J* = 7.8 Hz, H-3 and H-5 of C_6_H_5_), 7.87 (d, 2H, *J* = 7.8 Hz, H-2 and H-6 of C_6_H_4_), 10.00 (s, D_2_O exch., 1H, =NNH–), 10.79 (s, D_2_O exch., 1H, –CONH–); ^13^C NMR (DSMO-*d*_6_) δ ppm: 14.00, 55.20, 114.43, 114.54, 114.87, 115.02, 122.44, 128.14, 131.50, 133.82, 137.31, 145.00, 154.15; MS *m*/*z* [%]: 346 [(M + 2)^+^, 2.3], 344 [M^+^, 6.6], 121 [100].

##### (1*Z*,2*E*)-*N*-(4-Fluorophenyl)-2-(2-(4-methoxybenzoyl)hydrazono)propane hydrazonoyl chloride (**14e**)

Yellow powder (yield 77%), m.p.: 197–199 °C; IR (KBr, *ν* cm^−1^): 3320, 3252 (2NH), 1624 (C=O) and 1556 (C=N); ^1^H NMR (DMSO-*d*_6_) δ ppm: 2.38 (s, 3H, CH_3_), 3.84 (s, 3H, OCH_3_), 7.02 (d, 2H, *J* = 7.2 Hz, H-2 and H-6 of 4-FC_6_H_4_), 7.10 (t, 2H, *J* = 9 Hz, H-3 and H-5 of 4-FC_6_H_4_),7.33–7.37 (m, 2H, H-3, H-5 of OCH_3_C_6_H_5_), 7.91 (d, 2H, *J* = 8.7 Hz, H-2 and H-6 of OCH_3_C_6_H_5_,), 10.10 (s, D_2_O exch., 1H, =NNH–), 10.64 (s, D_2_O exch., 1H, –CONH–); ^13^C NMR (DSMO-*d*_6_) δ ppm: 13.61, 55.34, 113.33, 113.50, 115.04 (^3^*J*_F–C_ = 7.65 Hz), 115.42 (^2^*J*_F–C_ = 22.2 Hz) 115.85, 123.79, 125.68, 130.32, 140.16,155.56 (^1^*J*_F–C_ = 235 Hz), 161.93; MS *m*/*z* [%]: 364 [(M + 2)^+^, 0.9], 362 [M^+^, 2.7], 326 [100].

##### (1*Z*,2*E*)-*N*-(4-Chlorophenyl)-2-(2-(4-methoxybenzoyl)hydrazono)propane hydrazonoyl chloride (**14f**)

Yellow powder (yield 71%), m.p.: 200–203 °C; IR (KBr, *ν* cm^−1^): 3242–3320 (2NH), 1626 (C=O) and 1557 (C=N); ^1^H NMR (DMSO-*d*_6_) δ ppm: 2.37 (s, 3H, CH_3_), 3.84 (s, 3H, OCH_3_), 7.03 (d, 2H, *J* = 8.7 Hz, H-2 and H-6 of 4-ClC_6_H_4_),7.24–7.40 (m, 4H, Ar–H), 7.90 (d, 2H, *J* = 8.7 Hz, H-2 and H-6 of OCH_3_C_6_H_5_,), 10.24 (s, D_2_O exch., 1H, =NNH–), 10.68 (s, D_2_O exch., 1H, –CONH–); MS *m*/*z* [%]: 380 [(M + 2)^+^, 0.8], 378 [M^+^, 1.2], 135 [100].

##### (1*Z*,2*E*)-*N*-(4-Iodophenyl)-2-(2-(4-methoxybenzoyl)hydrazono)propanehydrazonoyl chloride (**14g**)

Yellow powder (yield 74%), m.p.: 195–196 °C; IR (KBr, *ν* cm^−1^): 3312, 3268 (2NH), 1659 (C=O) and 1582 (C=N); ^1^H NMR (DMSO-*d*_6_) δ ppm: 2.36 (s, 3H, CH_3_), 3.84 (s, 3H, OCH_3_), 7.03 (d, 2H, *J* = 9 Hz, H-2 and H-6 of 4-IC_6_H_4_), 7.16 (d, 2H, *J* = 9 Hz, H-3 and H-5 of 4-IC_6_H_4_), 7.58 (d, 2H, *J* = 8.7 Hz, H-3 and H-5 of 4-OCH_3_C_6_H_4_), 7.90 (d, 2H, *J* = 8.7 Hz, H-2 and H-6 of OCH_3_C_6_H_5_), 10.20 (s, D_2_O exch., 1H, =NNH–), 10.66 (s, D_2_O exch., 1H, –CONH–); MS *m*/*z* [%]: 472 [(M + 2)^+^, 0.3], 470 [M^+^, 1.1], 135 [100].

##### (1*Z*,2*E*)-2-(2-(4-Methoxybenzoyl)hydrazono)-*N*-(p-tolyl)propanehydrazonoyl chloride (**14h**)

Yellow powder (yield 70%), m.p.: 187–189 °C; IR (KBr, *ν* cm^−1^): 3257–3404 (2NH), 1624 (C=O) and 1555 (C=N); ^1^H NMR (DMSO-*d*_6_) δ ppm: 2.24 (s, 3H, CH_3_), 2.37 (s, 3H, CH_3_), 3.83 (s, 3H, OCH_3_), 7.03 (d, 2H, *J* = 8.7 Hz, H-2 and H-6 of 4-CH_3_C_6_H_4_), 7.08 (d, 2H, *J* = 8.7 Hz, H-3 and H-5 of 4-CH_3_C_6_H_4_), 7.22 (d, 2H, *J* = 8.4 Hz, H-3 and H-5 of 4-OCH_3_C_6_H_4_), 7.89 (d, 2H, *J* = 8.4 Hz, H-2 and H-6 of 4-OCH_3_C_6_H_4_), 10.06 (s, D_2_O exch., 1H, =NNH–), 10.68 (s, D_2_O exch., 1H, –CONH–); ^13^C NMR (DSMO-*d*_6_) δ ppm: 13.66, 20.24, 55.36, 113.35, 113.51, 113.85, 123.14, 125.72, 129.48, 129.60, 129.80, 130.33, 141.27, 161.91; MS *m*/*z* [%]: 360 [(M + 2)^+^, 1.1], 358 [M^+^, 3.2], 135 [100].

##### (1*Z*,2*E*)-2-(2-(4-Chlorobenzoyl)hydrazono)-*N*-phenylpropanehydrazonoyl chloride (**14i**)

White powder (yield 75%), m.p.: 222–224 °C; IR (KBr, *ν* cm^−1^): 3304, 3167 (2NH), 1653 (C=O) and 1543 (C=N); ^1^H NMR (DMSO-*d*_6_) δ ppm: 2.38 (s, 3H, CH_3_), 6.89 (t, 1H, *J* = 6.9 Hz, H-4 of –C_6_H_5_), 7.26–7.36 (m, 4H, H-2,H-3, H-5 and H-6 of –C_6_H_5_), 7.57 (d, 2H, *J* = 8.4 Hz, H-3 and H-5 of 4-ClC_6_H_4_), 7.90 (d, 2H, *J* = 8.4 Hz, H-2 and H-6 of 4-ClC_6_H_4_), 10.16 (s, D_2_O exch., 1H, =NNH–), 10.90 (s, D_2_O exch., 1H, –CONH–); ^13^C NMR (DSMO-*d*_6_) δ ppm: 13.72, 113.89, 118.50, 121.16, 126.47, 128.28, 129.16, 130.04, 132.47, 136.32, 143.45, 151.50; MS *m*/*z* [%]: 350 [(M + 2)^+^, 2.1], 348 [M^+^, 3.0], 312 [100].

##### (1*Z*,2*E*)-2-(2-(4-Chlorobenzoyl)hydrazono)-*N*-(4-fluorophenyl)propanehydrazonoyl chloride (**14j**)

Yellow powder (yield 79%), m.p.: 187–188 °C; IR (KBr, *ν* cm^−1^): 3325, 3187 (2NH), 1633 (C=O) and 1564 (C=N); ^1^H NMR (DMSO-*d*_6_) δ ppm: 2.37 (s, 3H, CH_3_), 7.10 (t, 2H, *J* = 8.7 Hz, H-3, H-5 of –FC_6_H_5_), 7.32–7.37 (q, 2H, *J* = 4.2, 4.8 Hz, H-2 and H-6 of –FC_6_H_5_), 7.57 (d, 2H, *J* = 8.4 Hz, H-3 and H-5 of 4-ClC_6_H_4_), 7.90 (d, 2H, *J* = 8.4 Hz, H-2 and H-6 of 4-ClC_6_H_4_), 10.18 (s, D_2_O exch., 1H, =NNH–), 10.89 (s, D_2_O exch., 1H, –CONH–); MS *m*/*z* [%]: 368 [(M + 2)^+^, 0.9], 366 [M^+^, 1.2], 139 [100].

##### (1*Z*,2*E*)-2-(2-(4-Chlorobenzoyl)hydrazono)-*N*-(p-tolyl)propanehydrazonoyl chloride (**14k**)

Yellow powder (yield 73%), m.p.: 205–207 °C; IR (KBr, *ν* cm^−1^): 3302, 3247 (2NH), 1667 (C=O) and 1584 (C=N); ^1^H NMR (DMSO-*d*_6_) δ ppm: 2.24 (s, 3H, CH_3_), 2.37 (s, 3H, CH_3_), 7.08 (d, 2H, *J* = 8.4 Hz, H-2 and H-6 of 4-CH_3_C_6_H_4_), 7.22 (d, 2H, *J* = 8.4 Hz, H-3 and H-5 of 4-CH_3_C_6_H_4_), 7.57 (d, 2H, *J* = 8.7 Hz, H-3 and H-5 of 4-ClC_6_H_4_), 7.90 (d, 2H, *J* = 8.7 Hz, H-2 and H-6 of 4-ClC_6_H_5_), 10.06 (s, D_2_O exch., 1H, =NNH–), 10.87 (s, D_2_O exch., 1H, –CONH–); ^13^C NMR (DSMO-*d*_6_) δ ppm: 14.00, 20.29, 113.88 (2C), 122.88 (2C), 128.22, 129.46, 129.90, 132.52(2C), 141.21, 150.00; MS *m*/*z* [%]: 364 [(M + 2)^+^, 5.0], 362 [M^+^, 7.6], 106 [100].

##### (1*Z*,2*E*)-2-(2-(4-Chlorobenzoyl)hydrazono)-*N*-(4-methoxyphenyl)propanehydrazonoyl chloride (**14l**)

Yellow powder (yield 76%), m.p.: 190–192 °C; IR (KBr, *ν* cm^−1^): 3326, 3184 (2NH), 1660 (C=O) and 1557 (C=N); ^1^H NMR (DMSO-*d*_6_) δ ppm: 2.36 (s, 3H, CH_3_), 3.71 (s, 3H, OCH_3_), 6.87 (d, 2H, *J* = 8.7 Hz, H-3 and H-5 of 4-OCH_3_C_6_H_4_), 7.26 (d, *J* = 8.7 Hz, 2H, H-2 and H-6 of 4-OCH_3_C_6_H_4_), 7.56 (d, 2H, *J* = 8.4 Hz, H-3 and H-5 of 4-ClC_6_H_4_), 7.89 (d, 2H, *J* = 8.4 Hz, H-2 and H-6 of 4-ClC_6_H_5_), 10.03 (s, D_2_O exch., 1H, =NNH–), 10.85 (s, D_2_O exch., 1H, –CONH–); MS *m*/*z* [%]: 380 [(M + 2)^+^, 5.8], 378 [M^+^, 8.6], 121 [100].

##### (1*Z*,2*E*)-2-(2-(Benzo[d]thiazole-2-carbonyl)hydrazono)-*N*-(4-chlorophenyl)propane hydrazonoyl chloride (**14m**)

Yellow powder (yield 77%), m.p.: 23–239 °C [35].

##### (1*Z*,2*E*)-*N*-(4-Bromophenyl)-2-(2-(3-methylbenzofuran-2-carbonyl)hydrazono)propane hydrazonoyl chloride (**14n**)

Yellow powder (yield 79%), m.p.: 235–237 °C [36].

#### 3.1.2. Synthesis of Compounds **16a**–**d**

A mixture of hydrazone **14a** or **14m** (1 mmol) and sodium benzene/toluenesulfinate **15a**,**b** (2 mmol) were refluxed in absolute ethyl alcohol for 10 h. The reaction mixture was left to cool then poured into ice-water. The solid product was filtered off, washed with water, dried and crystallized from EtOH/DMF to furnish the sulfones **16a**–**d**.

##### *N*'-((1*Z*,2*E*)-1-(2-(4-Chlorophenyl)hydrazono)-1-(phenylsulfonyl)propan-2-ylidene) benzohydrazide (**16a**)

Yellow powder (yield 70%), m.p.: 245–247 °C; IR (KBr, *ν* cm^−1^): 3228–3300 (2NH), 1639 (C=O), 1583 (C=N) and 1284, 1134 (SO_2_); ^1^H NMR (DMSO-*d*_6_) δ ppm: 2.49 (s, 3H, CH_3_), 6.91 (d, 2H, *J* = 8.4 Hz, H-2 and H-6 of 4-ClC_6_H_4_), 7.34 (d, 2H, *J* = 8.4 Hz, H-3 and H-5 of 4-ClC_6_H_4_), 7.54–7.84 (m, 6H, Ar–H), 7.95–7.99 (m, 4H, Ar–H), 11.72 (s, D_2_O exch., 1H, =NNH–), 14.58 (s, D_2_O exch., 1H, –CONH–); ^13^C NMR (DSMO-*d*_6_) δ ppm: 15.06, 115.87, 116.01, 125.00, 127.22, 128.48, 129.36, 129.52, 130.50, 132.27, 133.82, 135.19, 139.20, 141.38, 147.33, 165.28; MS *m*/*z* [%]: 456 [(M + 2)^+^, 4.6], 454 [M^+^, 11.8], 105 [100].

##### *N*'-((1*Z*,2*E*)-1-(2-(4-Chlorophenyl)hydrazono)-1-tosylpropan-2-ylidene)benzohydrazide (**16b**)

Yellow powder (yield 73%), m.p.: 237–239 °C; IR (KBr, *ν* cm^−1^): 3267–3310 (2NH), 1648 (C=O), 1577 (C=N) and 1290, 1134 (SO_2_); ^1^H NMR (DMSO-*d*_6_) δ ppm: 2.48 (s, 3H, CH_3_), 2.52 (s, 3H, CH_3_), 6.95 (d, 2H, *J* = 8.4 Hz, H-2 and H-6 of 4-ClC_6_H_4_), 7.35 (d, 2H, *J* = 8.4 Hz, H-3 and H-5 of 4-ClC_6_H_4_), 7.50–7.65 (m, 5H, Ar–H), 7.82 (d, 2H, *J* = 8.1 Hz, H-2 and H-6 of 4-CH_3_C_6_H_4_), 7.97 (d, 2H, *J* = 6.6 Hz, H-2 and H-6 of C_6_H_5_), 11.67 (s, D_2_O exch., 1H, =NNH–), 14.57 (s, D_2_O exch., 1H, –CONH–); MS *m*/*z* [%]: 470 [(M + 2)^+^, 2.6], 468 [M^+^, 6.4], 105 [100].

##### *N*'-((1*Z*,2*E*)-1-(2-(4-Chlorophenyl)hydrazono)-1-(phenylsulfonyl)propan-2-ylidene) benzo [*d*]thiazole-2-carbohydrazide (**16c**)

Yellow powder (yield 68%), m.p.: 240–242 °C; IR (KBr, *ν* cm^−1^): 3270–3311 (2NH), 1684 (C=O), 1590 (C=N) and 1289, 1137 (SO_2_); ^1^H NMR (DMSO-*d*_6_) δ ppm: 2.28 (s, 3H, CH_3_), 6.91 (d, 2H, *J* = 9 Hz, H-2 and H-6 of 4-ClC_6_H_4_), 7.28 (m, 1H, Ar–H), 7.37 (d, 2H, *J* = 9 Hz, H-3 and H-5 of 4-ClC_6_H_4_), 7.63–7.85 (m, 4H, Ar–H), 7.97 (d, 2H, *J* = 7.5 Hz, Ar–H), 8.26 (d, 2H, *J* = 7.5 Hz, Ar–H), 12.39 (s, D_2_O exch., 1H, =NNH–), 14.20 (s, D_2_O exch., 1H, –CONH–); MS *m*/*z* [%]: 513 [(M + 2)^+^, 2.0], 511 [M^+^, 4.4], 370 [100].

##### *N*'-((1*Z*,2*E*)-1-(2-(4-Chlorophenyl)hydrazono)-1-tosylpropan-2-ylidene)benzo[*d*] thiazole-2-carbohydrazide (**16d**)

Yellow powder (yield 70%), m.p.: 239–241 °C; IR (KBr, *ν* cm^−1^): 3274–3312 (2NH), 1682 (C=O), 1592 (C=N) and 1247, 1134 (SO_2_); ^1^H NMR (DMSO-*d*_6_) δ ppm: 2.22 (s, 3H, CH_3_), 2.37 (s, 3H, CH_3_), 6.91 (d, 2H, *J* = 8.4 Hz, H-2 and H-6 of 4-ClC_6_H_4_), 7.17–7.35 (m, 3H, Ar–H), 7.37 (d, 2H, *J* = 8.4 Hz, H-3 and H-5 of 4-ClC_6_H_4_), 7.51 (d, 1H, *J* = 8.1 Hz, Ar–H), 7.59–7.72 (m, 1H, Ar–H), 7.83 (d, 1H, *J* = 8.1 Hz, Ar–H), 8.25 (m, 2H, Ar–H), 12.38 (s, D_2_O exch., 1H, =NNH–), 14.17 (s, D_2_O exch., 1H, –CONH–); ^13^C NMR (DSMO-*d*_6_) δ ppm: 13.00, 21.50, 115.24, 116.13, 123.15, 124.01, 124.49, 125.15, 127.46, 128.62, 128.97, 129.73, 135.33, 136.18, 141.15, 142.47, 144.16, 144.60, 150.97, 152.64, 157.84, 167.35; MS *m*/*z* [%]: 527 [(M + 2)^+^, 2.0], 525 [M^+^, 4.5], 126 [100].

#### 3.1.3. Synthesis of Compounds **17a**,**b**

To a solution of the appropriate hydrazone **14c** or **14n** (1 mmol) in absolute ethyl alcohol (30 mL), piperidine (0.34 g, 4 mmol) and benzaldehyde (0.1 g, 1 mmol) were added. The reaction mixture was refluxed for 15 h. The precipitated product was filtered off while hot, washed with ethanol and dried. Recrystallization from EtOH/DMF gave compounds **17a** and **17b**, respectively.

##### *N*'-((1*Z*,2*E*,3*E*)-1-(2-(4-Iodophenyl)hydrazono)-4-phenyl-1-(piperidin-1-yl)but-3-en-2-ylidene)benzohydrazide (**17a**)

Yellow powder (yield 60%), m.p.: 210–212 °C; IR (KBr, *ν* cm^−1^): 3275–3320 (2NH), 1680 (C=O) and 1586 (C=N); ^1^H NMR (DMSO-*d*_6_) δ ppm: 1.56 (m, 6H, 3CH_2_ of piperidine), 3.21 (m, 4H, 2CH_2_ of piperidine), 6.81 (d, 2H, *J* = 8.7 Hz, Ar–H), 6.85 (s, IH, CH=CH), 6.91 (s, 1H, –CH=Ph), 7.07–7.40 (m, 8H, Ar–H), 7.44 (t, 1H, *J* = 7.5 Hz, Ar–H), 7.58–7.60 (m, 1H, Ar–H), 7.65 (d, 2H, *J* = 7.8 Hz), 8.39 (s, D_2_O exch., 1H, =NNH–), 10.39 (s, D_2_O exch., 1H, –CONH–); MS *m*/*z* [%]: 577 [M^+^, 6.7], 458 [100].

##### *N*'-((1*Z*,2*E*,3*E*)-1-(2-(4-Bromophenyl)hydrazono)-4-phenyl-1-(piperidin-1-yl)but-3-en-2-ylidene)-3-methylbenzofuran-2-carbohydrazide (**17b**)

Orange powder (yield 55%), m.p.: 224–226 °C; IR (KBr, ν cm^−1^): 3262–3318 (NH), 1679 (C=O) and 1593 (C=N); ^1^H NMR (DMSO-*d*_6_) δ ppm: 1.64 (m, 6H, 3CH_2_ of piperidine), 2.50 (s, 3H, CH_3_), 3.28 (m, 4H, 2CH_2_ of piperidine), 6.87 (s, 1H, –CH=CH–), 6.93 (d, 2H, *J* = 9 Hz, Ar–H), 7.13 (s, 1H, =CH–Ph), 7.18–7.56 (m, 7H, Ar–H), 7.59 (d, 2H, *J* = 7.5 Hz, Ar–H), 7.77 (d, 2H, *J* = 7.5 Hz, Ar–H), 8.54 (s, D_2_O exch., 1H, =NNH–), 10.58 (s, D_2_O exch., 1H, –CONH–); ^13^C NMR (DSMO-*d*_6_) δ ppm: 8.64, 23.95, 46.60, 71.37, 108.40, 111.44, 114.21, 114.37, 121.49, 126.57, 123.94, 127.41, 128.09, 128.76, 130.98, 131.15, 131.61, 135.61, 136.67, 141.32, 144.20, 146.67, 148.81, 152.73, 154.89; MS *m*/*z* [%]: 585 [(M + 2)^+^, 2.8], 585 [M^+^, 2.4], 175 [100].

### 3.2. Biological Evaluation

#### 3.2.1. Antimicrobial Activity

All strains were provided from culture collection of the Regional Center for Mycology and Biotechnology (RCMB), Al-Azhar University, Cairo, Egypt. Antibacterial and antifungal activities were expressed as the diameter of inhibition zones; Agar well diffusion method was used. Holes (1 cm diameter) were digger in the agar using sterile cork borer in sterile malt agar plates for fungi and sterile nutrient agar plates for bacteria, which had previously been uniformly seeded with tested microorganisms. The holes were filled by fungal filtrates (100 μL). Plates were left in a cooled incubator at 4 °C for one hour for diffusion and then incubated at 37 °C for tested bacteria and 28 °C for tested fungi. Inhibition zones developed due to active antimicrobial metabolites were measured after 24 h of incubation for bacteria and 48 h of incubation for fungi. Amphotericin B and ciprofloxacin were used as antifungal and antibacterial positive control, respectively. The experiment was performed in triplicate and the average zone of inhibition was calculated.

#### 3.2.2. Minimum Inhibitory Concentration

MIC was performed by a serial dilution technique described by Irobi *et al.* [52], starting with 100-mmol concentration of all compounds dissolved in 1 mL DMSO and then reduced by successive twofold dilutions of stock solution using a calibrated micropipette. Amphotericin B and ciprofloxacin were used as the reference compounds for fungi and bacteria; respectively. The final solutions concentrations were 125, 62.50, 31.25, 15.63, 7.81, 3.90, 1.95, 0.98, 0.49, 0.24 and 0.12 μmol/mL. The microtiter plates were incubated at 37 °C for tested bacteria and 28 °C for tested fungi and were readied using microplate reader after 24 h for bacteria and after 48 h for fungi. In each case, triplicate tests were performed and the average was taken as final reading. MIC was expressed as the lowest concentration inhibiting test organism’s growth [53].

#### 3.2.3. Antimycobacterial Activity

*M. tuberculosis* (RCMB 010126) strain was provided from culture collection of the Regional Center for Mycology and Biotechnology (RCMB), Al-Azhar University, Cairo, Egypt. The isolated *M. tuberculosis* (RCMB 010126) clone was cultivated under agitation on LB medium at 37 °C for 24 h. The antitubercular activity was expressed as the diameter of inhibition zones using agar well diffusion method and determination of MIC using serial dilution technique. Isoniazide and pyrazinamide were used as the reference drugs. The final solutions concentrations were 125, 62.50, 31.25, 15.63, 7.81, 3.90, 1.95, 0.98, 0.49, 0.24 and 0.12 μmol/mL. The zones of inhibition were analyzed after 72 h of incubation at 37 °C. Each test was repeated 3 times. MIC was expressed as the lowest concentration inhibiting test organism’s growth.

#### 3.2.4. *In Vitro* Cytotoxicity

A549 human lung cancer cells were grown in DMEM, supplemented with 10% heat inactivated FBS, 50 units/mL of penicillin and 50 g/mL of streptomycin and maintained at 37 °C in a humidified atmosphere containing 5% CO_2_. The cells were maintained as “monolayer culture” by serial subculturing. Cytotoxicity was determined using SRB method as previously described by Skehan *et al.* [54]. Exponentially growing cells were collected using 0.25% Trypsin-EDTA and seeded in 96-well plates at 1000–2000 cells/well in DMEM supplemented medium. After 24 h, cells were incubated for 72 h with various concentrations of the tested compounds. Following 72 h treatment, the cells will be fixed with 10% trichloroacetic acid for 1 h at 4 °C. Wells were stained for 10 min at room temperature with 0.4% SRB dissolved in 1% acetic acid. The plates were air dried for 24 h and the dye was solubilized with Tris-HCl for 5 min on a shaker at 1600 rpm. The optical density (OD) of each well was measured spectrophotometrically at 564 nm with an ELISA microplate reader (ChroMate-4300, Palm City, FL, USA).The IC_50_ values were calculated according to the equation for Boltzman sigmoidal concentration–response curve using the nonlinear regression fitting models (Graph Pad, Prism Version 5, GraphPad Software, Inc., San Diego, CA, USA).

## 4. Conclusions

In summary, the antimicrobial and antimycobacterial activities of halophenyl bis-hydrazones were evaluated. The results evidenced that compounds **14c**, **14g**, **16b**, **17a** and **17b** can be considered as broad spectrum antimicrobials against aspergillosis, Gram positive bacteria and Gram negative bacteria. Within the screened compounds, the latter five hydrazide/hydrazones with high lipophilicity showed the most promising antitubercular activity with MIC ranging from 1.95–7.81 μg/mL. The attractive results achieved with these analogs suggest that may be worth developing further in order to identify potential broad-spectrum antimicrobial and anti-TB chemotherapeutics. In addition, the generated QSAR models, performed to explore the structural requirements controlling the observed antibacterial properties, hinted that the biological activities were affected by jurs descriptors and shadow indices of the synthesized hydrazones.
